# On the Treatment of Rheumatic Fever

**Published:** 1864-04

**Authors:** Willoughby F. Wade

**Affiliations:** Senior Physician to the Queen’s Hospital, Birmingham


### ON THE TREATMENT OF RHEUMATIC FEVER.

By WILLOUGHBY F. WADE, M. D., M. R., C. P.

Senior Physician to the Queen's Hospital, Birmingham.

The few remarks I have to make refer chiefly to the treatment of rheumatic fever; still there are one or two points to which I should like to allude first.

Rheumatic Fever seems a more appropriate term than Acute Rheumatism, inasmuch as, by the consent of probably all pathologists, the disease is now esteemed to be an essential fever, rather than a pyrexia consequent upon, and therefore indicative of, local disorder. Most of us must have seen cases in which the febrile symptoms were well developed, whilst the joints remained little or not at all affected. Graves particularly notices this feature, which is also recognized by Latham and Fuller. Now, this is characteristic of idiopathic fevers; nevertheless, in rheumatic, as in typhoid, fevers, the pyrexia may be re-excited or maintained by an accession of new or an aggravation of old local disorder. This fact, as connected with continued fever, has been thrown into relief by Dr. Jenner—"the Dr. Jenner of our day," as Dr Watson so gracefully styles him. Attempts have been made to determine the exact nature of the *materies morbi rheumatici.* Lithic acid, lactic acid, and an undue preponderance of fibrin in the blood —hyperinosis, as it is termed—have each been claimants for this bad pre-eminence. Their respective titles to this position I shall not now occupy time in discussing, inasmuch as the question of the exact nature of the rheumatic poison, if such poison there be, is still, in my opinion, an open one.

With regard to the superabundance of fibrin in rheumatic fever, which I believe to be an admitted fact, it has recently acquired additional importance. We know how rare is delirium in this disease, whatever its severity. True, it is not unknown, but when present, it would seem to depend upon some peculiar and not well understood condition, and not to be proportioned to the intensity of the pyrexia simply, as in other fevers. Now, the researches of Drs. Charles Hood, Marcet, Erlenmeyer, and Hittorff seem to show that there is always a marked deficiency of fibrin in the blood of maniacs, either acute or chronic. A similar condition was found by Andral in idiopathic fevers, of which, when at all severe, delirium is so common a feature. Are we, then justified in suspecting that there is any connexion between the hyperinosis of rheumatic blood, and the infrequency of delirium in rheumatic fever? A therapeutic test would seem to show that when delirium does occur in this disease, the condition of the system approaches that of typhus. I have seen several well marked cases—one some years ago, which was treated, I forget how, but certainly not with stimulants. It terminated fatally: no marks of inflammation were found in the head. Three others have been under my own care during the past two years—one in the Queen's Hospital, surpervening on diarrhœa and attended with bedsore, which recovered perfectly under the use of wine in considerable quantity. The third case, which I saw at Dudley in consultation with Mr. Fereday, also supervened upon diarrhœa, and also recovered upon a freely stimulating plan. These three patients were females. A fourth case, in a man, likewise supervened on diarrhœa, and recovered after free stimulation. The occurrence of diarrhœa in those cases, and their recovery under a highly stimulating plan (recommended first by Dr. Todd), connect them with cases of what were termed by Dr. Marshall Hall the "hydrencephaloid" disease of children, of which diarrhœa is commonly the precursor, and which is also to be best met by stimulants.

This infantile disorder did not escape the acute observation of Dr. Gooch.

Two more cases have come under my notice since the preceding remarks were written. One was complicated with extensive pneumonia. Both supervened upon diarrhœa, and both recovered under the free use of stimulants.

This hyperinosis of rheumatic blood has other significant connexions. A similar peculiarity is exhibited by the blood of anæmic patients. Now, we have all heard of anæmia as a sequela and result of rheumatic fever, but we hear little or nothing of what I believe to be far more common—namely, rheumatic fever as a sequela of anæmia. A deficiency of red corpuscles is one of the most common prodromata of the malady. This fact has been concealed by two circumstances. In the first place, when large bleedings, low diet, and inefficient medication were employed in its treatment, with a consequent prolongation of the disease over months, instead of weeks or days, it seemed superfluous to inquire whether the patient had been previously anæmic. In the next place, the flushed countenance and hard full pulse of the fever disguise the ordinary symptoms of anæmia—pallor and feeble pulse,—which do not become manifest until the pyrexia has passed off, and are then attributed to its vehemence. We are often similarly misled in cases of chlorosis. When the patient is first seen, the face flushes with excitement, and the heart's action is stimulated by the same cause; and it is not till this simulation of health has subsided that we discover the well-known proofs of anæmia. I venture, then, to affirm, that in most cases of rheumatic fever we shall be able to elicit a previous history of that debility which is a common concomitant of deficincy in the red globules of the blood. And another feature of such blood—viz., its excessive proportion of fibrin—is, we know, a most constant if not essential characteristic of rheumatic blood.

I will now pass on to the treatment I adopt in cases of uncomplicated rheumatic fever—that is to say, the routine treatment; for I contend that whilst different cases may present differences requiring certain modifications, yet, on the whole, there are few diseases which are more constant to their respective typical forms, and, if we are to admit the propriety of routine treatment at all, we may safely include this complaint in the category of diseases which may be so dealt with.

I may state that for many years I have paid especial attention to this subject, and when I first went to the General Hospital as house-physican, now eleven years ago, I had, by the kindness of the then physicians to that institution, ample opportunity for employing the various plans recommended by authors.

The first thing to be done, then, is to put the patient in bed, and wrap up each joint in a thick layer of cotton wool, secured but not too tightly, by a tape or bandage. This answers two purposes: in the first place, it preserves the joint from pressure, and so removes one great source of pain and apprehension; in the next place, it preserves a uniform warm temperature of the joint favorable to perspiration, which, being highly acid, we suppose to contain either the actual *materies morbi* or else some of its secondary products, retention of which within the system can not be otherwise than prejudicial. Besides, the presence of this acid in the blood supplied to the joint we are led to believe to be the chief cause of the affection; its removal, therefore, even in small quantity, from the joint tends to cure the local disease, though it may produce but little effect upon the general symptoms. Dr. Todd recommends that a layer of oiled silk be wrapped outside the cotton wood; and he adds that, when removed, the wool will be found completely saturated with moisture. True, if we confine all the perspiration of twenty-four hours by means of the impervious coating, the wool will become moist, but this is no proof that there has been more fluid secreted than there would have been had it escaped as soon as formed; I think, indeed, we are likely to have less. However, if any one chooses to adopt this method, I would recommend them to add to the wool a quantity of finely powdered alkali (soda or potash).

Dr. Fuller relates some experiments bearing upon this point. He found that more speedy relief has been obtained by applying an opiated alkaline lotion to the joint than by warm water, solution of nitrate of potash, or simple alkaline solution. He goes on to say, "That the effect observed to follow these saline fomentations has not been due to accidental causes is manifest from the result of careful experiment; for, in order to guard against any source of fallacy, I selected fourteen instances in which corresponding joints were affected, and applied a fomentation of warm water to the one joint and an alkaline and opiate solution to the other, and almost uniformly the pain and inflammation continued in the former, and speedily subsided by the latter. Besides this, I have endeavored to ascertain whether the occurrence of inflammation may not be prevented in impregnating the parts with alkaline matter. In nine cases in which the knees and in severe cases in which the hands were in the first instance unaffected, a mixed saline and opiate solution was applied to one joint, and a simple water fomentation to the corresponding joint on the other side. In four of the former cases, and in two of the latter, the joint to which water only had been applied became inflamed, whilst in one instance only did inflammation occur and that to a very slight extent, in the joint which had been bathed in an alkaline and opiate solution."

These applications might, I think, be used more frequently as preventives of pericarditis. There seems, for example, to be an unusual tendency to pericarditis in rheumatic fever secondary to scarlatina. Of this form of disease I have seen five instances; in four of these there was pericarditis, complicated in two of them with pneumonia. The pericarditis was in the four cases, of unusual severity and importance. The fifth case, sister of one of the others, was the only instance of scarlatinal rheumatic fever in which spongio-piline saturated with a solution of bicarbonate of potash (half an ounce to eight ounces) was applied to the precordial region, and the only one in which pericarditis did not supervene.

I think the patient's comfort is best secured by a simple application (cotton wool), which does not involve a redisturbance of thepart, to the inflamed joints, and by letting the others alone. This being done, I administer a dose of calomel of from five to ten grains in combination with from thirty to sixty grains of the compound jalap powder. In a few hours this produces several copious evacuations containing much bile and causing a soothed and comfortable feeling as soon as the patient has recovered from the pain and fatigue consequent upon its operation. Indeed, a beneficial effect is oftentimes perceived by the patient some time before any ostensible results have ensued.

Independently of the value which experience awards to this plan as applied to rheumatic fever, we know that in other active febrile disorders a purge containing calomel relieves the systemic irritation in a marked degree. Besides, one of the commonest precursors of rheumatic fever is a yellowness of the sclerotics, which, as well as the irregular state of the bowels and unhealthy character of the motions, indicates the desirability of acting upon the hepatic system. This plan, indeed, has been adopted to the exclusion of all other medication. Dr. Chambers, who was a skilful as well as a fashionable practitioner, was in the habit of prescribing ten grains of calomel every night and a draught of salts and senna the following morning, as Dr. Latham informs us, with much success.

There is this reason against adopting it exclusively: the disturbance of the patient is very painful and distressing, and I think that after the free operation of the single dose, he may be spared all necessity for this disturbance by adopting another plan for three or four days, by which time his movements will be comparatively unrestricted. Dr. Latham points out that whilst Dr. Chambers' method is styled "the purgative plan," yet its purpose is achieved by calomel and purgatives conjointly. "The purgatives," he says, "would not answer the end without the calomel; of that I am quite certain. Neither would the calomel answer without the purgatives, unless it produced of itself ample evacuations from the bowels. It is probable, in short, that the medical efficacy of the plan resides essentially in the calomel; in calomel, however, not as *mercury*, but as itself—calomel." But it often happens that a patient has taken a quantity of purgative medicine before we are called to him. I am so convinced of the efficacy of the calomel that even under those circumstances I still administer it; but, instead of combining it with a purgative, I added ten or fifteen grains of Dover's powder, which acts as a calmative and promotes perspiration, without diminishing, even if it does not ncrease, the cholagogue properties of the calomel.

[*To be continued.*]

